# Sequential analysis and its applications to neuromorphic engineering

**DOI:** 10.3389/fnins.2025.1735027

**Published:** 2026-01-09

**Authors:** Shivaram Mani, Saeed Afshar, Travis Monk

**Affiliations:** International Centre for Neuromorphic Systems, The MARCS Institute, Western Sydney University, Sydney, NSW, Australia

**Keywords:** applied statistics, event camera, event sensor, hypothesis testing, likelihood ratio, neuromorphic computing, sequential analysis, threshold crossing

## Abstract

**Introduction:**

Neuromorphic circuits operate by comparing fluctuating signals to thresholds. This operation underpins sensing and computation in both neuromorphic architectures and biological nervous systems. Rigorous analysis of such systems is rarely attempted because the statistical tools to study them are both inaccessible and largely unknown to the neuromorphic community.

**Methods:**

We offer a gentle introduction to one such tool, sequential analysis, a classical framework that addresses a particular class of threshold-crossing problems. We define the formal problem analyzed in sequential analysis and present Abraham Wald's elegant methodology for solving it.

**Results:**

We then apply this framework to three examples in neuromorphic engineering, demonstrating how it can serve as a benchmark, proxy model, and design tool. Our introduction is understandable without prior training in probability or statistics.

**Discussion:**

Sequential analysis provides the statistical limits of circuit performance, tractable abstractions of complex circuit behavior, and constructive rules for circuit design. It establishes rigorous statistical baselines for evaluating hardware. It links low-level circuit parameters to observable dynamics, clarifying the computational role of neuromorphic architectures. By translating performance goals into optimal thresholds and design parameters, it offers principled prescriptions that go beyond empirical tuning.

## 1 Introduction

Neuromorphic engineering develops hardware and software systems based on the structure and function of nervous systems. Its principal goal is to design efficient, adaptive, and robust computation beyond conventional digital architectures (Christensen et al., 2022; Indiveri et al., 2011; Mead, 1989). Many neuromorphic devices encode and process information using discrete “spikes” or “events” (Mahowald, 1992). These spikes are typically generated when an internal variable, e.g., a voltage, crosses a threshold.

Threshold crossing problems are well-studied in statistics. They arise whenever a fluctuating process is compared to one or more boundaries (Monk et al., 2015, 2024, 2014; Monk and van Schaik, 2021, 2022; Gold and Shadlen, 2007; Monk and van Schaik, 2020; Urzay et al., 2023). One example is a spiking neuron whose membrane potential exceeds the firing threshold (Mani et al., 2025; Shadlen and Shohamy, 2016; Kira et al., 2015). Another is a pixel in an event-based sensor whose voltage crosses “on” or “off” thresholds to generate events (Gallego et al., 2022). Statistics provides powerful tools for analyzing these problems (Doob, 1953; Lai, 2009b; Tartakovsky et al., 2014; Taylor and Karlin, 1984). But those tools remain largely inaccessible to the neuromorphic community. Much of the statistics literature is written in abstract mathematical language, which obscures its applicability to neuromorphic systems.

In this study, we introduce sequential analysis, a classical statistical framework for threshold crossing problems, to the neuromorphic community (Wald, 1944). Sequential analysis was pioneered by Abraham Wald (Wald, 1944, 1947) to study optimal decision-making when data arrive over time. It provides exact results for decision accuracy, decision times, and optimal thresholds. We demonstrate that these results translate naturally to neuromorphic circuits. In the Methods, we present the formal problem of sequential analysis and its solution. Then, in the Results, we demonstrate how sequential analysis functions as a **benchmark**, **proxy model**, and **design tool** in three neuromorphic applications. We deliberately avoid technical jargon to make the derivations accessible to readers without a background in probability theory. All prerequisite materials for our derivations are available as Supplementary material online. Threshold crossing problems provide a rigorous and intuitive lens for benchmarking, interpreting, and designing neuromorphic architectures. By formalizing how evidence is accumulated toward a decision, sequential analysis provides a principled framework for interpreting the behavior and computational role of both neuromorphic circuits and biological neurons.

## 2 Materials and methods

If any step in the following derivation is unclear, Supplementary material walks through the underlying principles in plain language. No prior background in statistics is required to understand this material; only the SI.

**Figure 1:**
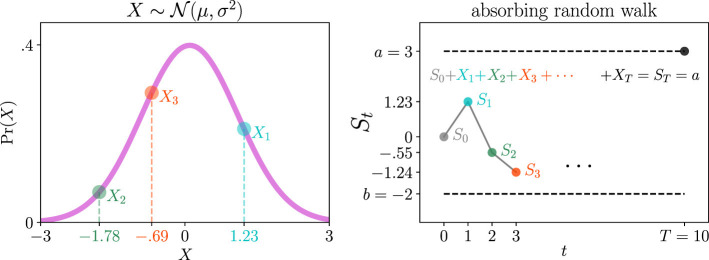
Schematic of a sequential analysis problem. **(Left)** We observe realizations of a random variable *X*, one per time step. *X* is assumed to be independent and identically distributed at every time step. In this example, Pr(*X*) is a normal distribution with mean μ and variance σ^2^ (magenta trace). Three samples from the first three time steps, *X*_1_, *X*_2_, and *X*_3_, are shown in cyan, green, and red, respectively. **(Right)** Sequential analysis considers the cumulative sum *S*_*t*_ of those realizations of *X*. It compares *S*_*t*_ to two constant absorbing barriers *a* and *b* (e.g., on and off thresholds). While *b* < *S*_*t*_ < *a*, we observe new realizations of *X* and continue adding them to the cumulative sum. We want to find the probabilities that the sum hits either barrier before the other and the distribution of the number of realizations *T* required to hit it. In this example, *S*_*T*_ = *a* and *T* = 10 time steps (black dot, upper-right of the panel).

Figure 1 illustrates a sequential analysis problem (Wald, 1944; Monk and van Schaik, 2020). Let *S*_*t*_ represent the cumulative sum of *t* realizations of a random variable *X*~Pr(*X*). At each time step, we observe a new realization of *X* and add it to the sum of all previous realizations, *S*_*t*−1_. Thus, *S*_*t*_ is a random walk that changes by *X* at each time step. The key assumption of sequential analysis is that *X* is independent and identically distributed (i.i.d.) for all time steps.

The left panel in Figure 1 plots an example distribution Pr(*X*) as a normal distribution with mean μ and variance σ^2^ (magenta trace). The left panel also shows three realizations drawn from Pr(*X*) at the first three time steps, shown in cyan, green, and red, respectively. The right panel in Figure 1 shows that we add each realization of *X* to the sum of all previous observations.

The right panel in Figure 1 also illustrates that sequential analysis assumes the random walk *S*_*t*_ to be between two constant thresholds *b* and *a* (horizontal dashed black lines). In this example, *b* = −2, *a* = 3, and the initial sum is *S*_0_ = 0. As long as *b* < *S*_*t*_ < *a*, we continue making new observations of *X* and adding them to *S*_*t*_ (gray, cyan, green, and red dots). When *S*_*t*_ crosses either threshold (e.g., threshold *a* at random time *T* = 10, black dot, right panel), we stop the random walk. One goal of sequential analysis is to obtain the probability of crossing one threshold before the other, i.e., Pr(*S*_*T*_ = *a*) and Pr(*S*_*T*_ = *b*). Another goal is to find the conditional distributions of *T*, Pr(*T*∣*S*_*T*_ = *a*) and Pr(*T*∣*S*_*T*_ = *b*).

Abraham Wald derived threshold crossing probabilities and conditional time distributions from a martingale (Wald, 1944; Lai, 2009b; Doob, 1953). Consider the conditional expectation:


$$𝔼\left[e^{S_{t}h}\phi_{X}{\left(h\right)}^{-t}|S_{t-1}\right],$$

where ϕ_*X*_(*h*) is the MGF of *X* and *h* is its (real) independent variable. Wald's analysis requires that the MGF be well-defined within a specific domain of *h*. It also requires regularity conditions, e.g., integrability and boundedness. In most practical applications of sequential analysis (including the examples we will consider here), these conditions hold.

We can quickly show that this conditional expectation is a martingale. Notice that ϕ_*X*_(*h*) is a deterministic function. Moreover, *t* is just the number of time steps that have elapsed up to a given time. Since neither is random, we can pull them out of the conditional expectation:


$$𝔼\left[e^{S_{t}h}\phi_{X}{\left(h\right)}^{-t}|S_{t-1}\right]=\phi_{X}{\left(h\right)}^{-t}𝔼\left[e^{S_{t}h}|S_{t-1}\right].$$

Insert *S*_*t*_ = *S*_*t*−1_ + *X*_*t*_ in the right-hand side:


$$=\phi_{X}{\left(h\right)}^{-t}𝔼\left[e^{S_{t-1}h}e^{X_{t}h}|S_{t-1}\right].$$

Given *S*_*t*−1_, the term exp(*S*_*t*−1_*h*) is not random, so pull it out:


$$=\phi_{X}{\left(h\right)}^{-t}e^{S_{t-1}h}𝔼\left[e^{X_{t}h}|S_{t-1}\right].$$

Since the *X* are i.i.d., *X*_*t*_ is independent of *S*_*t* − 1_:


$$=\phi_{X}{\left(h\right)}^{-t}e^{S_{t-1}h}𝔼\left[e^{X_{t}h}\right].$$

Recognize the expectation as the MGF of *X* and simplify:


$$\begin{array}{l} =e^{S_{t-1}h}\phi_{X}{\left(h\right)}^{-\left(t-1\right)}. \end{array}$$

Comparing this expression to our original conditional expectation, we observe that it forms a martingale:


$$𝔼\left[e^{S_{t}h}\phi_{X}{\left(h\right)}^{-t}|S_{t-1}\right]=e^{S_{t-1}h}\phi_{X}{\left(h\right)}^{-(t-1)}.$$

Next, we write this martingale as a conservation statement. Take the expectations of both sides and apply the law of total expectation:


$$\begin{array}{l} 𝔼\left[e^{S_{t}h}\phi_{X}{\left(h\right)}^{-t}\right]=𝔼\left[e^{S_{t-1}h}\phi_{X}{\left(h\right)}^{-\left(t-1\right)}\right]. \end{array}$$

By induction:


$$\begin{array}{l} 𝔼\left[e^{S_{t}h}\phi_{X}{\left(h\right)}^{-t}\right]=𝔼\left[e^{S_{0}h}\phi_{X}{\left(h\right)}^{0}\right]=e^{S_{0}h}, \end{array}$$

assuming that *S*_0_ is known (i.e., not random) and *t* begins at 0. This equation states that the expectation of the quantity $e^{S_{t}h}\phi_{X}{\left(h\right)}^{-t}$ is conserved throughout this stochastic process.

Doob's optional stopping theorem states that a randomly stopped martingale is still a martingale (Doob, 1953). Thus, insert the random stopping time *T* for *t*:


$$\begin{array}{l} 𝔼\left[e^{S_{T}h}\phi_{X}{\left(h\right)}^{-T}\right]=e^{S_{0}h}. \end{array}$$ (1)

Equation 1 is known as the fundamental identity in sequential analysis (Wald, 1944; Tartakovsky et al., 2014). Wald derived threshold crossing probabilities and conditional time distributions from it (Wald, 1944, 1947). The reason we can extract so much threshold crossing information from it is that it is valid for all values of *h*. We extract our desired quantities by choosing special values of *h* and inserting them into Equation 1 (Monk and van Schaik, 2020).

**Figure 2:**
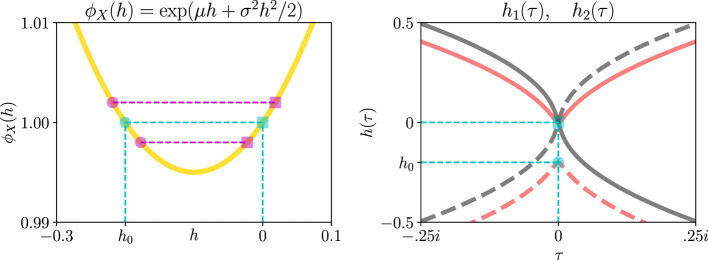
Special values of *h* extract threshold crossing probabilities and times from Wald's martingale. **(Left)** Under weak assumptions, the MGF of a random variable is convex and crosses 1 at exactly two values of *h* (cyan markers and lines). All MGFs cross 1 at *h* = 0 (square cyan marker), so that crossing is trivial and we discard it. The other crossing is nontrivial, and we use it to compute threshold-crossing probabilities. Furthermore, under those weak assumptions, the MGF crosses one twice in the *neighborhood* of 1 (magenta markers and lines). Then the logarithm of the MGF has two real roots in *h* in the neighborhood of 0. So for imaginary τ, the logarithm of the MGF has two complex roots. **(Right)** Those two complex roots *h*_1_(τ) and *h*_2_(τ) for the MGF in the left panel. We use them to calculate conditional threshold crossing times. Solid traces represent one root, and dashed traces represent the other. Red and gray traces represent the real and imaginary parts of those roots, respectively. When τ = 0, those complex roots pass through the MGF crossings of 1 (cyan markers and lines).

Figure 2 visualizes the special values of *h* that we insert into the martingale. First, we calculate the probabilities of crossing the threshold. The left panel of Figure 2 plots the MGF of *X*, given the same distribution as shown in the left panel of Figure 1. Given weak assumptions (Wald, 1944), ϕ_*X*_(*h*) is convex and crosses 1 at exactly two values of *h* (cyan lines and markers). Since all MGFs equal 1 at *h* = 0 by definition, we discard one of those crossings because it provides no useful information (square cyan marker, left panel Figure 2). The second crossing occurs at a nontrivial value *h* = *h*_0_≠0 (circle cyan marker). Inserting *h*_0_ into Equation 1:


$$\begin{array}{l} 𝔼\left[e^{S_{T}h_{0}}\phi_{X}{\left(h_{0}\right)}^{-T}\right]=𝔼\left[e^{S_{T}h_{0}}\right]=e^{S_{0}h_{0}}. \end{array}$$

For compact notation, let α ≡ Pr(*S*_*T*_ = *a*) and β ≡ Pr(*S*_*T*_ = *b*). Split the expectation, conditional on crossing either threshold first:


$$𝔼\left[e^{S_{T}h_{0}}\right]=𝔼\left[e^{S_{T}h_{0}}|S_{T}=a\right]\alpha +𝔼\left[e^{S_{T}h_{0}}|S_{T}=b\right]\beta =e^{S_{0}h_{0}}.$$

Given that *S*_*T*_ is *a* or *b*, the terms in the conditional expectations are just constants (i.e., not random):


$$\begin{array}{l} e^{ah_{0}}\alpha +e^{bh_{0}}\beta =e^{S_{0}h_{0}}. \end{array}$$

The process will cross either threshold in finite time (Wald, 1944), so we can insert β = 1−α and rearrange:


$$\begin{array}{l} \alpha =\frac{e^{S_{0}h_{0}}-e^{bh_{0}}}{e^{ah_{0}}-e^{bh_{0}}}. \end{array}$$ (2)

The right panel of Figure 2 shows the values of *h* that we insert into Equation 1 to obtain the conditional characteristic functions (CCFs) of threshold crossing times. Under weak assumptions (Wald, 1944), ϕ_*X*_(*h*) has two real-valued crossings of a horizontal line in the *neighborhood* of 1, and not just at 1 (magenta lines and markers, left panel Figure 2). So its logarithm has two real roots in *h* in that neighborhood. Then for imaginary τ, −logϕ_*X*_(*h*) = τ has two complex roots *h*_1_(τ) and *h*_2_(τ):


$$\phi_{X}\left(h_{1}\left(\tau \right)\right)=\phi_{X}\left(h_{2}\left(\tau \right)\right)=e^{-\tau }.$$

Insert *h*_1_(τ) into Equation 1:


$$𝔼\left[e^{S_{T}h_{1}}\phi_{X}{\left(h_{1}\right)}^{-T}\right]=𝔼\left[e^{S_{T}h_{1}}e^{\tau T}\right]=e^{S_{0}h_{1}}.$$

Split the expectation, conditional on hitting either threshold first:


$$\alpha 𝔼\left[e^{S_{T}h_{1}}e^{\tau T}|S_{T}=a\right]+(1-\alpha )𝔼\left[e^{S_{T}h_{1}}e^{\tau T}|S_{T}=b\right]=e^{S_{0}h_{1}}.$$

Given which threshold was crossed first, exp(*S*_*T*_*h*_1_) is not random. Pull it out of the conditional expectations:


$$\alpha e^{ah_{1}}𝔼\left[e^{\tau T}|S_{T}=a\right]+(1-\alpha )e^{bh_{1}}𝔼\left[e^{\tau T}|S_{T}=b\right]=e^{S_{0}h_{1}}.$$

Recognize the conditional expectations as the CCFs of *T*, ψ_*T*|*a*_(τ) and ψ_*T*|*b*_(τ):


$$\alpha e^{ah_{1}}\psi_{T|a}\left(\tau \right)+\left(1-\alpha \right)e^{bh_{1}}\psi_{T|b}\left(\tau \right)=e^{S_{0}h_{1}}$$

Now, insert *h*_2_(τ) into Equation 1, repeat the same argument, and we have a system of two equations:


$$\begin{array}{l} \alpha e^{ah_{1}}\psi_{T|a}\left(\tau \right)+\left(1-\alpha \right)e^{bh_{1}}\psi_{T|b}\left(\tau \right)=e^{S_{0}h_{1}};\\ \alpha e^{ah_{2}}\psi_{T|a}\left(\tau \right)+\left(1-\alpha \right)e^{bh_{2}}\psi_{T|b}\left(\tau \right)=e^{S_{0}h_{2}}. \end{array}$$

Since we have two equations, we can rearrange for both CCFs:


$$\begin{array}{l} \psi_{T|a}\left(\tau \right)=\frac{e^{S_{0}h_{1}\left(\tau \right)}e^{bh_{2}\left(\tau \right)}-e^{S_{0}h_{2}\left(\tau \right)}e^{bh_{1}\left(\tau \right)}}{\alpha \left(e^{ah_{1}\left(\tau \right)}e^{bh_{2}\left(\tau \right)}-e^{ah_{2}\left(\tau \right)}e^{bh_{1}\left(\tau \right)}\right)};\\ \psi_{T|b}\left(\tau \right)=\frac{e^{S_{0}h_{2}\left(\tau \right)}e^{ah_{1}\left(\tau \right)}-e^{S_{0}h_{1}\left(\tau \right)}e^{ah_{2}\left(\tau \right)}}{\left(1-\alpha \right)\left(e^{ah_{1}\left(\tau \right)}e^{bh_{2}\left(\tau \right)}-e^{ah_{2}\left(\tau \right)}e^{bh_{1}\left(\tau \right)}\right)}. \end{array}$$ (3)

By the law of total expectations, the marginal CF of T is as follows:


$$\begin{array}{l} \psi_{T}\left(\tau \right)=\alpha \psi_{T|a}\left(\tau \right)+\left(1-\alpha \right)\psi_{T|b}\left(\tau \right). \end{array}$$

When 𝔼[*X*] = 0, Equation 2 is undefined. In this special case, ϕ_*X*_(*h*) only crosses 1 once, at the trivial value *h* = *h*_0_ = 0. We circumvent this issue by taking the limit of Equation 2 as *h*_0_ → 0:


$$\begin{array}{l} \underset{h_{0}\to 0}{\lim}\alpha =\frac{S_{0}-b}{a-b}. \end{array}$$

Alternatively, we can find a simpler martingale that allows us to find α in this special case:


$$𝔼\left[S_{t}|S_{t-1}\right]=𝔼\left[S_{t-1}+X_{t}|S_{t-1}\right]=S_{t-1}+𝔼\left[X_{t}\right]=S_{t-1},$$

and we have another martingale. Writing it as a conservation statement and invoking Doob's optional stopping theorem:


$$\begin{array}{l} 𝔼\left[S_{T}\right]=𝔼\left[S_{0}\right]=S_{0}. \end{array}$$

Split the expectation conditional on which threshold was crossed:


$$\alpha 𝔼\left[S_{T}|S_{T}=a\right]+(1-\alpha )𝔼\left[S_{T}|S_{T}=b\right]=S_{0}.$$

Evaluate the conditional expectations and rearrange:


$$\begin{array}{l} \alpha a+\left(1-\alpha \right)b=S_{0}\;\to \;\alpha =\frac{S_{0}-b}{a-b}, \end{array}$$

and we recover the same expression.

Wald's analysis is exact when *S*_*T*_ hits either threshold exactly, i.e., when there is never any threshold overshoot. Generally speaking, *S*_*T*_ can overshoot thresholds more when the variance of *X* is high. If the threshold overshoot is large with respect to the distance between the thresholds, then Wald's analysis can yield inaccurate estimates of threshold crossing probabilities and times. The literature reports techniques to estimate and/or bound threshold overshoot in order to obtain more accurate approximations of these quantities (Lai, 2009a). Since this paper is an introduction to sequential analysis, these techniques are beyond the scope of this study. We can often reduce barrier overshoot by defining the time step to be extremely brief so that *S*_*t*_ changes only slightly from one time step to the next. This approach is valid for many neuromorphic applications, in which a continuous quantity fluctuates between thresholds.

## 3 Results

We now show how to apply sequential analysis in neuromorphic engineering, including worked examples.

### 3.1 Characterizing idealized event sensor pixel noise

**Figure 3:**
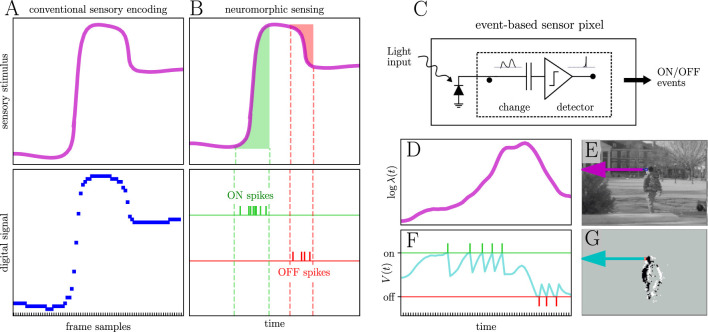
Event sensors represent visual data differently than conventional cameras. **(A)** Conventional sensors convert an analog signal (top plot) into a series of frames that uniformly discretize the signal in space and time (bottom plot). **(B)** Neuromorphic sensors efficiently represent that analog signal (top plot) with precisely timed events (raster plots, bottom) when it increases (ON, green) or decreases (OFF, red). **(C)** Simplified circuit diagram of a neuromorphic vision sensor pixel. **(D)** Illustrative log-intensity of photons over time log λ(*t*) on the highlighted pixel in **(E)**. **(F)** Corresponding voltage trajectory *V*(*t*) (cyan trace) for the highlighted neuromorphic pixel in panel G. *V*(*t*) approximates the change in log photon intensity from **(D)**. *V*(*t*) is compared to on (green) and off (red) thresholds. Threshold crossings generate events that mark the microsecond at which the log photon intensity changed beyond the threshold. When either threshold is crossed, *V*(*t*) is reset to the other threshold. Collectively, the panels demonstrate key advantages of neuromorphic sensing: microsecond temporal resolution, sparse output proportional to scene dynamics, and in-pixel analog preprocessing that reduces bandwidth and energy consumption.

Figure 3 illustrates key differences between conventional and neuromorphic vision sensors. Panel A is a schematic of a visual scene representation by a conventional camera. Conventional cameras discretize an analog signal (i.e., incident light, top plot) uniformly across space (by pixels) and time (by frames). They output a series of digitized frames comprising pixel values that describe the light they observed at uniformly spaced moments in time (panel A, bottom plot). Panel B shows how neuromorphic vision sensors (e.g., event sensors) represent a scene. Event sensor pixels generate events in response to changes in light intensity, typically by monitoring changes in log intensity or voltage (Gallego et al., 2022). When the integrated change exceeds a certain positive threshold, an ‘on event' is generated (green shaded area and raster plot, panel B). Conversely, if the change is sufficiently negative, an ‘off event' is triggered (red shaded area and raster plot, panel B). Output events can occur within microseconds of a change in light intensity.

Figure 3C is a diagram illustrating how an event sensor pixel achieves such remarkable temporal precision. A photodiode transduces photons and charges a capacitor. The charging current is proportional to the change in the log intensity (logλ(*t*) of the incoming photons. Panel D shows a hypothetical log intensity of photons incident on a single pixel from the image of panel E. Panel F illustrates that the event sensor pixel then compares the voltage across the capacitor to on (green horizontal line) and off (red horizontal line) thresholds. When the voltage exceeds either threshold, the pixel generates a corresponding event. The green and red raster plots in panel F present hypothetical event output of a single pixel from the neuromorphic ‘image' in panel G. The circuitry implementing this comparison and event generation is not shown in panel C. Event sensors can output data at different rates depending on the scene that they are filming. If nothing in the scene is moving with respect to the sensor, its output events are sparse, and its resulting output data rate can be very low; vice versa. For example, the background of the scene illustrated in panel E is not moving with respect to the event sensor. Thus, event sensor pixels with a static background in their field of view will output few events, as shown in panel G. In contrast, conventional cameras output the same amount of data regardless of the scene.

Theoretically, if nothing in a scene changes, the event sensor should not output any events. However, in practice, event pixels generate ‘noisy' events even in a static scene with no true intensity change (Padala et al., 2018). Since there is no change in intensity, the light incident on a pixel during a timestep does not vary (regardless of how we define a timestep). Therefore, the key assumption of sequential analysis (i.i.d. timesteps) is met. We can then use sequential analysis to characterize the statistics of those noisy events, at least for idealized pixels. Even if sequential analysis does not accurately model the circuitry of a real event sensor pixel, it provides a benchmark for comparing the statistical properties of noisy events from a real event sensor.

**Figure 4:**
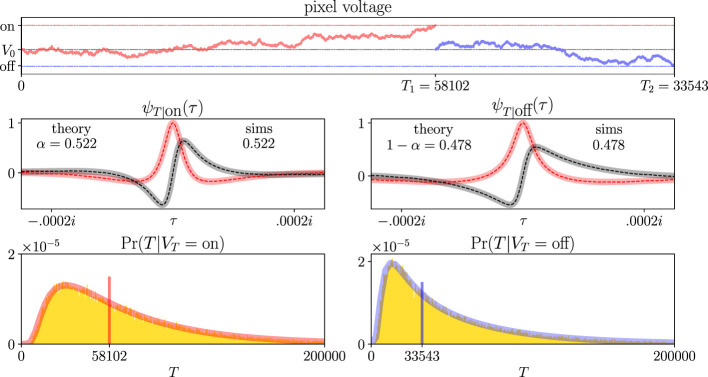
Noisy events from idealized event sensor pixels receiving constant light intensity is a sequential analysis problem. **(Top row)** Schematic of a single event sensor pixel's voltage fluctuating between an on and off threshold. If we assume that the pixel receives constant light intensity and the change in pixel voltage on a time step is i.i.d. We want to find the probability that the pixel generates an on or off event. The conditional waiting time distributions are used to do so. Two example voltage-path realizations are shown: one ultimately crosses the on threshold (red trace, upper dashed line) and the other crosses the off threshold (blue trace, lower dashed line). The waiting times of both paths are shown on the *x*-axis. **(Middle row)** Theoretical (solid traces) and simulation (dashed traces) CCFs and threshold crossing probabilities are practically identical. Real (pink/red) and imaginary (gray/black) parts of CCFs are plotted separately. **(Bottom row)** Analogous to the middle row, but with conditional probability distributions instead of CCFs. The red and blue traces are effectively inverse Fourier transforms of the CCFs above. The gold histograms are simulation results for conditional waiting times at threshold crossings. The two voltage-path realizations from the top row are plotted as samples from those distributions (red and blue bars). Simulation curves reflect 100,000 independent trials; sampling variance is below the line thickness at the plotted scale.

The top plot in Figure 4 considers an idealized event sensor pixel as a sequential analysis problem. Let the cumulative sum *S*_*t*_ represent the voltage of the pixel *V*_*t*_ at time *t*. Let the change in the sum *X* be the change in the pixel voltage Δ*V* on a time step. To keep our calculations simple, let ΔV~N(μ,σ). The thresholds *a* and *b* directly map to the on and off thresholds of the pixel. The top plot also shows two example realizations of the voltage path. One crosses the on threshold first (red trace and dashed line), and the other crosses the off threshold first (blue trace and dashed line). The waiting times for both voltage-path realizations are shown on the *x*-axis.

First, we calculate the probability that a pixel generates an on or off event first, i.e., threshold crossing probabilities. The MGF of Δ*V* is as follows:


$$\begin{array}{l} \phi_{\Delta V}\left(h\right)=\exp\left(\mu h+\sigma^{2}h^{2}/2\right). \end{array}$$

This MGF was plotted in the left panel of Figure 2. ϕ_Δ*V*_(*h*) crosses one twice, once at zero and again at a non-trivial value:


$$\begin{array}{l} \exp\left(\mu h_{0}+\sigma^{2}h_{0}^{2}/2\right)=1\;\to \;h_{0}=-2\mu /\sigma^{2}. \end{array}$$

Inserting *h*_0_ into Equation 2, we determine the probability that the pixel will generate an on event before an off event. Put in some example parameter values *a* = 3, *b* = −2, *V*_0_ = 0, μ = 10^−5^, and σ = 10^−2^:


$$\begin{array}{l} \alpha =\frac{e^{V_{0}h_{0}}-e^{bh_{0}}}{e^{ah_{0}}-e^{bh_{0}}}=\frac{1-e^{0.4}}{e^{-0.6}-e^{0.4}}\approx .522. \end{array}$$

Next, we calculate waiting-time CCFs for noisy on- and off-events. For imaginary τ, −logϕ_Δ*V*_(*h*) = τ has two complex roots *h*_1_(τ) and *h*_2_(τ) (right panel, Figure 2). Obtaining their expressions is straightforward:


$$\begin{array}{l} -\left(\mu h+\sigma^{2}h^{2}/2\right)=\tau \;\to \;h\left(\tau \right)=\frac{-\mu \pm \sqrt{\mu^{2}-2\sigma^{2}\tau }}{\sigma^{2}}. \end{array}$$

One root, say *h*_1_(τ), is given by one sign in the ± symbol, say +. The other root *h*_2_(τ) is given by the other sign. The right panel in Figure 2 plots these two complex roots. Inserting them into Equations 3, we find the waiting time CCFs of the idealized pixel's noisy events.

The middle row of Figure 4 plots these CCFs for the parameter values stated above (thick solid traces). Pink and gray traces plot the real and imaginary parts of the CCFs, respectively. We also print the threshold crossing probabilities α and 1−α. These panels also compare simulation results with theoretical results. We ran 100,000 independent simulations of this idealized event sensor pixel with the parameter values stated above. We saved which threshold was crossed first and how many time steps the process took to reach it. Then we computed the Fourier transforms of our resulting waiting-time distributions to compare them with our theoretical CCFs. The reported numbers in the panels indicate very strong agreement between our calculated and simulated threshold-crossing probabilities. The dashed red and black traces in the middle panels show that our simulated CCFs very closely match our theoretical CCFs. Since we assumed that the pixel voltage changes only very slightly at each time step, the voltage is extremely unlikely to overshoot either threshold appreciably. So Wald's analysis is practically exact, and we ran enough simulations to converge on his solution.

The bottom row of Figure 4 shows that we can recover conditional probability distributions of *T* from those CCFs. For example, we find Pr(*T*|*V*_*T*_ = on) from ψ_*T*|on_(τ) via the inverse Fourier transform:


$$\begin{array}{l} \text{Pr}\left(T|V_{T}=\text{on}\right)=\frac{1}{2\pi }\int_{\mathbb{R}}e^{-\tau T}\psi_{T|\text{on}}\left(\tau \right)d\tau . \end{array}$$

Pr(*T*|*V*_*T*_ = off) is found analogously. The red and blue traces in the bottom row show the conditional probability distributions calculated from the CCFs in the middle row. The gold bars are histograms of our simulation results. Again, we see a very strong match between Wald's analysis and our simulation results. The two example voltage-path realizations from the top row are also displayed as samples from their corresponding conditional waiting-time distributions.

We can construct other waiting time CFs from ψ_*T*|*a*_(τ) and ψ_*T*|*b*_(τ). For example, let *A* represent the waiting time until a pixel generates an on event. Let *B* represent the number of off events that occur while we wait for the on event. Since each threshold crossing is an i.i.d. trial, the CCF of *A* given *B* is:


$$\begin{array}{l} \psi_{A|B}\left(\tau \right)=\psi_{T|b}{\left(\tau \right)}^{B}\psi_{T|a}\left(\tau \right). \end{array}$$

We can calculate the CF of *A* from this CCF:


$$\begin{array}{l} \psi_{A}(\tau )=𝔼\left[e^{\tau A}\right]=𝔼\left[𝔼\left[e^{\tau A}|B\right]\right]=𝔼\left[\psi_{A|B}(\tau )\right]\\ \;=𝔼\left[\psi_{T|b}{\left(\tau \right)}^{B}\psi_{T|a}(\tau )\right]. \end{array}$$

We evaluate this expectation by noting that *B*~Geom(1−α):


$$\begin{array}{l} 𝔼\left[\psi_{T|b}{\left(\tau \right)}^{B}\psi_{T|a}\left(\tau \right)\right]=\overset{\infty }{\underset{B=0}{\sum }}\psi_{T|b}{\left(\tau \right)}^{B}\psi_{T|a}\left(\tau \right)\alpha^{B}\left(1-\alpha \right), \end{array}$$

then recognizing the sum as a geometric series:


$$\begin{array}{l} \psi_{A}\left(\tau \right)=\frac{\left(1-\alpha \right)\psi_{T|a}\left(\tau \right)}{1-\alpha \psi_{T|b}\left(\tau \right)}. \end{array}$$

### 3.2 Leaky current-integrating node given SPAD input

**Figure 5:**
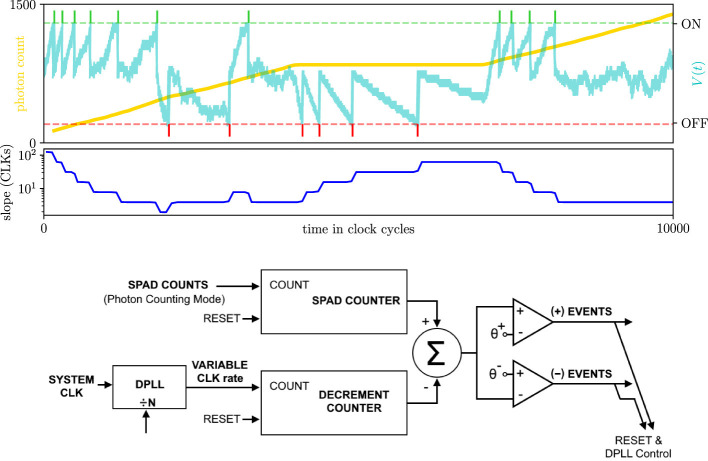
Diagram and operation of a leaky capacitive node that integrates SPAD sensor input. **(Top plot)** A single-photon avalanche diode (SPAD) converts each detected photon (the gold trace is the cumulative photon count, left y-axis) into identical current impulses that charge a capacitive node. The voltage across this node *V*(*t*) (cyan trace, right *y*-axis) leaks through a constant conductance and decays at a rate *m* between photon arrivals. Each photon arrival causes an instantaneous voltage jump of *u* in *V*(*t*). Two programmable comparators monitor *V*(*t*): an upper (on) threshold and a lower (off) threshold. When *V*(*t*) crosses the on threshold, an on event is emitted, and vice versa. After each event, *V*_*t*_ is reset between the thresholds. Notice that on events are more frequent as the photon count increases, and off events are more frequent when the photon count stagnates. **(Middle plot)** The leak rate *m* is decreased or increased after each on or off event, respectively (blue trace). This adaptive change implements a refractory-like gain control mechanism that balances the event rate between the two thresholds. **(Bottom plot)** Circuit diagram of the SPAD and capacitive node.

Figure 5 shows another hardware implementation of a sequential analysis problem (Delic and Afshar, 2020). A single-photon avalanche diode (SPAD) operated in Geiger mode converts every detected photon into a precisely timed electrical impulse. Early SPAD-event processing circuits illustrate this principle (Afshar et al., 2020). Efficient real-time processing of SPAD array data is the subject of active investigation (Gyongy et al., 2020; Delic and Afshar, 2024). The gold trace in the top plot of Figure 5 is a hypothetical cumulative count of photons detected by a SPAD (left *y*-axis, top plot). When that SPAD feeds a current-integrating node with a finite leak conductance, the node voltage *V*(*t*) executes a biased random walk. *V*(*t*) jumps up by a fixed amount *u* upon each photon arrival and drifts down with constant slope *m* between arrivals (Delic and Afshar, 2024; Morrison et al., 2020). The cyan trace in the top plot is a realization of that random walk, given the observation of photons (right *y*-axis, top plot). When *V*(*t*) crosses an on or off threshold (dashed green and red lines in the top plot), an event is generated (shown in green and red raster plots), and *V*(*t*) is reset between these points. Sequential analysis can thoroughly and tractably analyze this circuit.

The tractability of sequential analysis is a powerful feature. Absorption probabilities and times are expressed as functions of the problem's input parameters. Thus, if we change some parameter values upon threshold crossing, Wald's methodology remains applicable for analyzing future threshold crossings. For example, say we change the value of the decay rate *m* after every threshold crossing. We could increase or decrease it depending on which threshold is crossed to achieve a habituation-like gain-control mechanism. The blue trace in the middle plot of Figure 5 is a realization of *m* when we implement such a rule. Every time an on event is generated, the slope decreases, and vice versa. Thus, every event biases *V*(*t*) to hit the off threshold on the next random walk. In Figure 5, notice that an increasing photon count initially causes the current-integrating node to frequently generate on events. However, as *m* decreases, the on events habituate, and we begin to observe off events. Then, when the photon count stagnates, we observe the reverse effect. We could achieve a similar gain-control effect through other mechanisms. Upon crossing the threshold, we change the values of the thresholds to bias future crossings analogously. The circuit at the bottom of Figure 5 is a diagram of how to implement these types of feedback mechanisms in hardware. Whichever mechanism(s) we use, sequential analysis tells us how to compute threshold-crossing probabilities and times.

Define a ‘time step' to be the waiting time until a photon arrival, including the arrival itself. We model photon arrival to the SPAD as a Poisson process with rate λ. Then the waiting time until photon arrival *E* is exponentially distributed; *E* ~ Exp(λ). While waiting for the photon to arrive at the SPAD, the node voltage decays linearly at a rate *m* < 0. When a photon arrives, its voltage bumps up by a constant amount *u* > 0. Thus, the change in the node's voltage over a time step is as follows:


$$\begin{array}{l} X=mE+u. \end{array}$$

The MGF of *X* is as follows:


$$\begin{array}{c} \phi_{X}\left(h\right)=\phi_{mE}\left(h\right)\phi_{u}\left(h\right)=\phi_{E}\left(mh\right)\phi_{u}\left(h\right)\\ =e^{uh}\frac{\lambda }{\lambda -mh}\;\text{for }mh<\lambda . \end{array}$$

Again, we begin by calculating threshold crossing probabilities. It is noteworthy that we cannot find a closed-form expression for the non-trivial crossing at ϕ_*X*_(*h*_0_) = 1. We could numerically evaluate that crossing, but instead, we will employ an approximation. Assume that 𝔼[*X*]≈0 so that *h*_0_≈0. Taylor expanding the exponential:


$$\begin{array}{l} e^{uh_{0}}\frac{\lambda }{\lambda -mh_{0}}\approx \left(1+uh_{0}+u^{2}h_{0}^{2}/2\right)\frac{\lambda }{\lambda -mh_{0}}=1. \end{array}$$

Rearranging for *h*_0_:


$$\begin{array}{l} h_{0}\approx \frac{-2\left(m+\lambda u\right)}{\lambda u^{2}}. \end{array}$$

Equation 2 is very sensitive to the value of *h*_0_ used because *h*_0_ appears in exponentials. Thus, our approximation for *h*_0_ must be very accurate to yield accurate approximations of threshold crossing probabilities. We can enhance the accuracy of our approximation by adding more terms to the Taylor expansion of the exponential. Or we can revert to a numerical solver to achieve sufficient accuracy for Wald's analysis. Whatever method we use to obtain *h*_0_, threshold crossing probabilities are then given by Equation 2.

Next, we obtain waiting time CCFs by finding two complex roots *h*_1_(τ) and *h*_2_(τ) to the equation $\phi_{X}\left(h\right)=e^{-\tau }$:


$$\begin{array}{l} \left(1+uh+u^{2}h^{2}/2\right)\frac{\lambda }{\lambda -mh}=e^{-\tau }. \end{array}$$

Rearranging, we find that *h*(τ) is given by the quadratic equation:


$$\begin{array}{l} h\left(\tau \right)=\pm \frac{\sqrt{{\left(\lambda u+me^{-\tau }\right)}^{2}-2\lambda^{2}u^{2}\left(1-e^{-\tau }\right)}}{\lambda u^{2}}-\frac{me^{-\tau }}{\lambda u^{2}}-\frac{1}{u}. \end{array}$$

Again, *h*_1_(τ) is given by one sign in the ± symbol, and *h*_2_(τ) by the other. Inserting *h*_1_(τ) and *h*_2_(τ) into Equations 3, we find the waiting time CCFs to threshold crossing.

**Figure 6:**
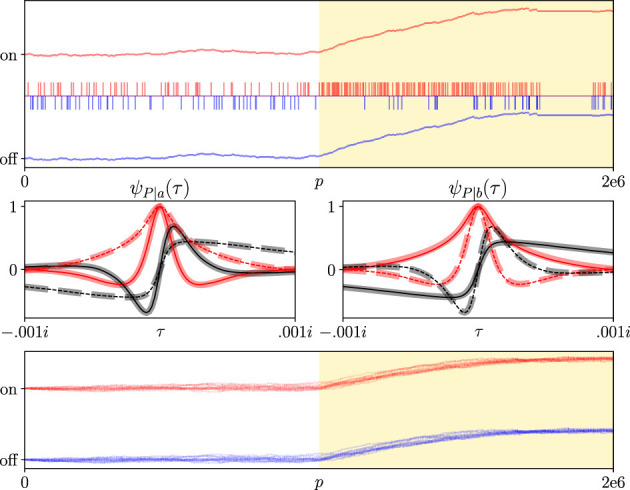
A SPAD sensor feeding inputs to a thresholded capacitive node is a sequential analysis problem. **(Top row)** Output spikes and thresholds of a capacitive node given SPAD sensor input. The SPAD sensor transduces Poisson-distributed photons into electrical impulses. Those impulses are accumulated by a capacitive node. When an impulse arrives, the node's voltage bumps up by an amount *u* = 0.1. As no impulses arrive, the voltage decays linearly at a rate of *m* = −0.00999. The voltage is bounded by two thresholds (red and blue traces), and the node produces on and off events (green and red raster plots) when either threshold is crossed. Both thresholds increase or decrease by 0.1 when the on or off threshold is crossed, respectively. Halfway through the simulation, the photon rate increases from λ = 0.1 to λ = 0.102 (yellow shaded area). As event frequency increases, off events become rare. The thresholds increase accordingly and stabilize at new, higher values when on and off events occur at similar rates. Middle row: CCFs of the number of photons required to cross the on (left panel) or off (right panel) threshold. CCFs and simulation results are plotted in a manner analogous to those in Figure 4. Each panel plots two CCFs, one with thresholds at –5 and 15 (solid traces) and the other with thresholds at –15 and 5 (dashed traces). **(Bottom row)** Threshold evolution of the capacitive node from 1,000 independent simulations identical to the top row. Thresholds were initialized at –10 and 10 with an initial voltage set to *V*_0_ = 0 in all simulations. Simulation curves reflect 100,000 independent trials; sampling variance is below line thickness at the plotted scale.

Figure 6 applies Wald's analysis to the circuit diagram at the bottom of Figure 5. We achieved gain control for the circuit by adjusting the on and off thresholds, depending on which threshold was crossed. The top row of Figure 6 shows one example of how the thresholds evolve over a single trial of 2e6 seconds (red and blue traces). We initialized *V*_0_ = 0 and the on and off thresholds at 10 and −10, respectively. Both thresholds were increased or decreased by 0.1, depending on which was crossed. Our other parameter values were *u* = 0.1, *m* = −0.00999, and λ = 0.1 (so 𝔼[*X*]≈0). Halfway through the simulation, we slightly increased the photon intensity from 0.1 to 0.102 photons per second (yellow shaded background, top plot). The timings of on- and off-threshold crossings are represented as red and blue raster plots, respectively. For the first half of the simulation, on and off events are approximately equally frequent, so thresholds do not appreciably change. Then, when λ increases, some events become significantly more frequent. Both thresholds increase, then balance each other at new values.

The middle row of Figure 6 plots CCFs of the number of photons required to hit either threshold, and for particular parameter values. It is directly analogous to the middle row of Figure 4. The only difference between the middle rows of Figures 4, 6 show that we plot two CCFs in each panel instead of one. Each CCF was plotted for different threshold values. The solid trace CCFs used threshold values of *a* = 15 and *b* = −5, and the dashed trace CCFs used *a* = 5 and *b* = −15. For all CCFs we used *V*_0_ = 0, λ = 0.1, and *u* = 0.1. We set *m* = λ*u*+10^−5^ to ensure that 𝔼[*X*]≈0 for all CCFs. The middle row of Figure 6 shows that Wald's CCFs are sensitive to threshold values. So every time the capacitive node spikes and we change the threshold values, those CCFs can change appreciably.

The bottom row of Figure 6 plots the evolution of the capacitive node's on and off thresholds over 1,000 independent trials. As in the top row, we changed the rate of incoming photons from λ = 0.1 to λ = 0.102 halfway through each trial (yellow-shaded background). Each time the node spiked, we adjusted both thresholds, as in the top row, depending on which threshold was crossed. Even a small 2% increase in the photon rate biases the node voltage to cross the on threshold with a much higher probability and in a much shorter time. Since both thresholds increase with each event from the node (and vice versa), both thresholds increase soon after the rate of incoming photons increases. Eventually, the thresholds saturate and stabilize around new values.

Recall that our definition of a time step was the waiting time until a photon arrival, including the arrival itself. Therefore, the random variables of our CCFs are the *number of photon arrivals* required to cross one threshold or the other. In Figure 6, notice that the random variable in the CCFs (middle row) and the x-axes of the top and bottom plots are numbers of photons (*P*), and not time *T*. We can easily switch random variables from *P* to *T*. Upon threshold crossing, *P* and *T* are linearly related:


$$\begin{array}{l} uP-mT=a\text{ or }b\;\to \;T=uP/m-\left(a\text{ or}b\right)/m. \end{array}$$

So the CCFs of *T* are:


$$\begin{array}{l} \psi_{T|a}\left(\tau \right)=\psi_{P|a}\left(\frac{u\tau }{m}\right)e^{a\tau /m};\;\psi_{T|b}\left(\tau \right)=\psi_{P|b}\left(\frac{u\tau }{m}\right)e^{b\tau /m}. \end{array}$$

### 3.3 Hypothesis testing

Sequential analysis is directly applicable to hypothesis testing (Wald and Wolfowitz, 1948). If we have two competing hypotheses *H*_0_ (the “null hypothesis”) and *H*_1_ (the “alternative hypothesis”). We observe data that support one hypothesis or the other until we accumulate sufficient evidence to accept one and reject the other with a specified confidence level. For example, imagine an SPAD sensor receiving photons at a rate λ that can only be one of two values λ_0_ or λ_1_. We can set our null hypothesis to be that λ = λ_0_ and our alternative hypothesis to be λ = λ_1_. We observe the output of the SPAD sensor until we conclude, with a confidence level, that the photon rate is one value or the other.

**Figure 7:**
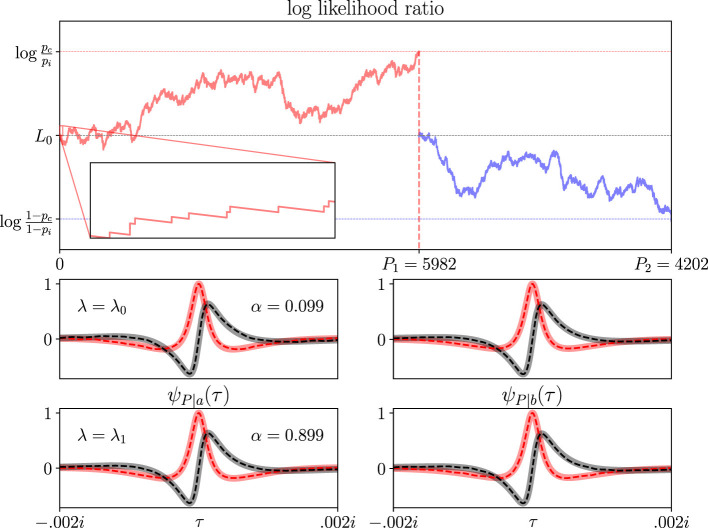
The sequential probability ratio test is a famous example of a sequential analysis problem. **(Top plot)** The sum *S*_*t*_ is the log likelihood *L*_*t*_ of data under two competing hypotheses. In this example, the hypotheses are the photon arrival rates λ_0_ and λ_1_. Two example paths of *L*_*t*_ are plotted, one crossing threshold *a* (red) and the other *b* (blue). Thresholds map to the probabilities of correct and incorrect hypothesis detection *p*_*c*_ and *p*_*i*_ (*y*-axis). When *L*_*t*_ crosses a threshold, the test accepts the corresponding hypothesis. The inset is a magnification showing the behavior of *L*_*t*_ given the first few photons. **(Bottom plots)** CCFs of the number of photons required for threshold crossing, ψ_*P*|*a*_(τ) (left column) and ψ_*P*|*b*_(τ) (right column). The top CCFs assume a photon rate of λ_0_ and the bottom CCFs assume a photon rate of λ_1_. Interpretation of these panels is directly analogous to all other CCFs presented in earlier figures. In all plots, λ_0_ = 0.1, λ_1_ = 0.102, *p*_*c*_ = 0.9, and *p*_*i*_ = 0.1. Notice that α ≈ *p*_*c*_ or *p*_*i*_, depending on which hypothesis was true. Simulation curves reflect 100,000 independent trials; sampling variance is below line thickness at the plotted scale.

The top plot in Figure 7 frames sequential analysis as an online algorithm for hypothesis testing. This algorithm is called the sequential probability ratio test (Wald and Wolfowitz, 1948) that has been applied across many disciplines (Li and Kulldorff, 2010; Kulldorff et al., 2011; Wang and Wan, 2017; Gold and Shadlen, 2007). Let the sum *S*_*t*_ be the log-likelihood ratio *L*_*t*_ of the data, given that either hypothesis is true. For example, we calculate *L*_*t*_ for a Poisson rate of incoming photons by taking the log of the ratio of Poisson distributions:


$$\begin{array}{l} L_{t}=\log\frac{\mathrm{Pr}\left(P|\lambda_{1}\right)}{\mathrm{Pr}\left(P|\lambda_{0}\right)}=P\left(\log\lambda_{1}-\log\lambda_{0}\right)-\left(\lambda_{1}-\lambda_{0}\right)t, \end{array}$$

for *P* photons in time *t*. The inset in the top plot shows how *L*_*t*_ increases by a constant amount upon photon arrival and decays linearly between arrivals. While *b*<*L*_*t*_<*a*, we continue observing new data points because we cannot accept either hypothesis with sufficient confidence. Then, when *L*_*t*_ crosses either threshold, we accept the corresponding hypothesis. Two example paths of *L*_*t*_ are plotted in Figure 7, one crossing the upper threshold (red path) and the other the lower threshold (blue path).

The thresholds *a* and *b* map beautifully to the probabilities of correct and incorrect hypothesis detection *p*_*c*_ and *p*_*i*_:


$$\begin{array}{l} a\approx \log p_{c}/p_{i};\;b\approx \log\left(1-p_{c}\right)/\left(1-p_{i}\right). \end{array}$$

Thus, before we start the test, we decide on ‘acceptable' errors: accepting the hypothesis *H*_0_ when *H*_1_ is true, and vice versa. Then we simply calculate the thresholds that we should use for the test to achieve those probabilities. In Figure 7, we set *p*_*c*_ = 0.9 and *p*_*i*_ = 0.1. One reason that the sequential probability ratio test is a very popular hypothesis testing algorithm is that it enjoys a significant optimality property. No other test can achieve the same or better *p*_*c*_ with a lower expected number of samples (Wald and Wolfowitz, 1948). When samples are expensive, e.g., testing the efficacy of a new drug that is expensive to manufacture, this optimality property is very valuable.

Our sequential probability ratio test on Poisson-distributed photons is identical to our application on the current-integrating node, given SPAD sensor input from the previous subsection. The linear decay between photon arrivals (what we called *m*) is (λ_1_−λ_0_) here. The voltage bumps upon photon arrivals (what we called *u*) are (logλ_1_−logλ_0_) here. Therefore, the capacitive node from the previous subsection **implements a sequential probability ratio test for two particular photon intensities**. The value of the photon intensities in the test is implied by the slope of the decay between photon arrivals and the amount its voltage bumps up upon photon arrivals:


$$\begin{array}{l} \;m=\lambda_{0}-\lambda_{1},\;u=\log\lambda_{1}-\log\lambda_{0}\\ \to \;\lambda_{0}=m+\frac{me^{u}}{1-e^{u}},\;\lambda_{1}=\frac{me^{u}}{1-e^{u}}. \end{array}$$

The thresholds of the capacitive node **define probabilities of correct and incorrect hypothesis detection of the test**. Thus, when we changed thresholds after each threshold crossing in the previous subsection, we implicitly changed those detection probabilities. The output spikes of the node are its assertions that it accepts one photon rate over another. We can simply reuse the mathematical analysis from the previous subsection for this sequential probability ratio test.

The bottom half of Figure 7 plots four waiting time CCFs that we obtained from Equation 3. More specifically, they are CCFs for the number of photons *P* required to cross either threshold. Again, real and imaginary parts of the CCFs are given by the pink/red and gray/black traces, respectively. Again, solid traces are theoretical results from Equation 3 and dashed traces are Fourier transforms of simulated waiting times until threshold crossing. In all plots, we set the photon rates to be λ_0_ = 0.1 and λ_1_ = 0.102. The CCFs in the left column are ψ_*P*|*a*_(τ) and the CCFs in the right column are ψ_*P*|*b*_(τ). The top CCFs were evaluated under hypothesis *H*_0_, i.e., the photon rate was λ = λ_0_. The bottom CCFs were evaluated under hypothesis *H*_1_. We print threshold crossing probabilities in the left panels. Notice that α≈0.1 and α≈0.9 are in close agreement with our desired probabilities of correct and incorrect detection *p*_*c*_ and *p*_*i*_.

## 4 Discussion

Threshold-crossing problems, such as sequential analysis, provide a rigorous framework for evaluating and/or interpreting neuromorphic architectures. We have illustrated how the same mathematical framework can play three different roles, depending on its context. First, sequential analysis can serve as a **benchmark** to compare against hardware. Similar to the Carnot engine in thermodynamics, it does not need to model real devices to be useful. Second, it can serve as a **proxy model** that makes circuit behavior tractable. By abstracting away transistor-level details, sequential analysis offers a tractable model that maps circuit parameters to dynamics. Third, it can serve as a **design tool** that prescribes optimal circuit behavior. Framing circuits as statistical decision-makers turns parameter selection into a principled mapping from problem specification to circuit design. Together, these three roles establish sequential analysis as a benchmark, proxy model, and design tool for neuromorphic hardware within a unified statistical language.

Our first application, noisy event pixels, illustrates the value of sequential analysis as a **benchmark**. Real pixels generate spurious events even under constant illumination (Lichtsteiner et al., 2008; iniVation, 2020; Hamilton et al., 2014). The statistics of those events are difficult to model in detail because of device mismatch and circuit-level complexity. Sequential analysis does not attempt to reproduce those details. Instead, it provides the ideal statistical baseline for fluctuations, defining what noise would look like if the pixel were to follow a perfectly tractable random process. This role is directly analogous to that of the Carnot engine in thermodynamics. No real physical engine achieves it, but it defines the efficiency ceiling and establishes a rigorous language for comparison. In the same way, sequential analysis supplies neuromorphic engineers with an ideal standard against which measured device behavior can be evaluated. Deviations from this benchmark are then informative rather than merely problematic.

Our second application, adaptive dynamics in neuromorphic circuits, illustrates the value of sequential analysis as a **proxy model**. Circuit behavior is often shaped by many interdependent parameters at the transistor level, making tractable analysis impossible. Sequential analysis does not replicate those low-level mechanisms. Instead, it provides a simplified statistical model in which adaptation appears as a shift in decision thresholds or effective time constants. This abstraction connects measurable input–output behavior to the circuit's underlying computational role. Sequential analysis provides a tractable stand-in that reveals how circuit parameters drive observable dynamics.

Our third application, decision-making circuits, illustrates the value of sequential analysis as a **design tool** (Burri et al., 2014; Woods et al., 2019). When circuits are viewed as statistical decision-makers, problem requirements such as tolerable error rates map directly onto circuit parameters, such as thresholds and decay rates. Sequential analysis provides the formal framework that defines this mapping, making design choices constructive rather than empirical. Under the assumptions of sequential analysis, hardware decision times inherit valuable optimality properties (Wald and Wolfowitz, 1948). This perspective also makes the computational function of a circuit node transparent. For example, a current-integrating node can be interpreted as implementing an online sequential probability ratio test on SPAD input. Each output event is its probabilistic assertion that the incident photon intensity belongs to one hypothesis rather than another. Threshold values specify the confidence level of those assertions. Sequential analysis, therefore, provides both design prescriptions and rigorous probabilistic interpretations of circuit behavior.

While powerful, sequential analysis rests on restrictive assumptions that limit its direct applicability. Classical formulations require independent and identically distributed observations. In practice, neuromorphic signals rarely meet this criterion. Inputs are often time-varying, reflecting both stimulus dynamics and adaptive circuit responses. They are also correlated across space and time, violating the independence assumptions of Wald's classical approach. Moreover, optimal sequential tests assume that the likelihood ratio between competing hypotheses can be written down explicitly. In practice, this explicit representation is rarely available. The probability distributions of real inputs are often too complex to admit closed-form likelihoods, e.g., due to device mismatch. These challenges do not invalidate sequential analysis, but they highlight the need for extensions that adapt the framework to more realistic conditions.

The assumption of stationarity is incompatible with sensory streams, where input rates fluctuate due to both external stimuli and circuit-level adaptation. Sequential analysis can still be applied by invoking the time-rescaling theorem (Brown et al., 2002; Haslinger et al., 2010), which transforms an inhomogeneous Poisson process into a homogeneous one of unit rate. This transformation preserves the statistics of event timing while removing the nonstationarity. So we can apply classical sequential methods in the rescaled time domain. If we also had an estimate of the inhomogeneous intensity (Ng and Murphy, 2019), we could invert our results to return to the original time domain. Circuits that integrate changing input rates can still be interpreted within a sequential framework, but with temporal variability absorbed into the rescaling.

The assumption of independence is also violated in practice, since neuromorphic signals are often correlated across space and time. These dependencies break the classical proofs of optimality that rely on the i.i.d. structure of Wald's original framework. However, the sequential paradigm itself does not require independence. It only requires that we can quantify how new evidence modifies the decision statistic. Correlations can be incorporated directly into the likelihood function when the joint distribution is known or approximated. Even when exact models are unavailable, structured approximations can preserve the essential behavior of the test statistic. Correlation complicates the math, but the core idea of accumulating evidence toward a threshold remains valid.

A further assumption is the absence of threshold overshoot: increments are implicitly taken to be small enough that the process crosses the boundary exactly rather than leaping past it. Real neuromorphic circuits do not obey this constraint. In practice, overshoot does not invalidate the sequential framework; it alters only the mapping between the physical voltage trajectory and the effective statistical test. Standard corrections are available: boundary-adjustment factors derived from renewal theory, first-passage approximations for jump processes, or continuous-time formulations in which the hazard rate characterizes crossings without requiring perfect boundary contact. These provide engineering countermeasures that preserve the utility of sequential analysis even when threshold crossings occur with finite overshoot.

Although our derivations rely on i.i.d. and stationary increments, their role in the three case studies is as a principled reference rather than a literal description of hardware or biology. For the idealized event-pixel example, the assumption isolates the fundamental stochastic mechanism underlying threshold crossings; real pixels introduce temporal correlations, drift, and device-level variability, but these effects serve as structured deviations from the baseline predicted by sequential analysis rather than contradictions. For the LIF node driven by SPAD-like input, the model already includes leaky dynamics and thus diverges from strict i.i.d. behavior; here, sequential analysis provides an analytically tractable approximation that captures the correct scaling of crossing statistics even when the microscopic increments are not perfectly independent. In the SPRT case study, non-i.i.d. evidence streams simply alter the effective log-likelihood update without compromising the decision-theoretic framework. Across all three examples, the idealized assumptions provide a clean baseline that clarifies how each system behaves when higher-order correlations, drift, or non-stationarities are added. This makes deviations interpretable and allows the sequential analysis predictions to function as a benchmark rather than a surrogate for full hardware realism.

Our contribution is conceptual rather than device-specific. We establish how classical sequential analysis provides analytically tractable predictions for threshold-crossing behavior and decision dynamics, and we demonstrate this across three distinct neuromorphic contexts. The case studies serve as proof of principle, showing how the framework can be integrated into existing modeling and design workflows. The simulations validate the theory under controlled conditions. Measurements of event-sensor noise often show deviations from the idealized model used in our first case study, including illumination-dependent drift and heavier-tailed inter-event statistics. These discrepancies are expected: sequential analysis defines the baseline fluctuations in the absence of such circuitry-specific effects, and real data typically sit above this baseline in predictable ways. Hardware evaluation is, therefore, a natural next step for future work.

Although our case studies focus on single nodes, the same framework extends naturally to larger SNNs. Threshold tuning in multi-layer networks is often heuristic, whereas sequential analysis provides explicit predictions for firing rates, false-alarm probabilities, and detection delays. These quantities can be propagated through a network because each layer filters and transforms the distribution observed by the previous one. This makes sequential analysis a principled complement to existing tuning methods: it provides analytical targets for threshold setting and clarifies how architectural changes affect the statistics of threshold events.

Sequential analysis occupies a distinct position relative to existing neuromorphic modeling approaches. Circuit-level models, such as mixed-signal simulations, differential-equation descriptions of neurons, or large-scale numerical SNN frameworks, emphasize numerical fidelity but rarely yield closed-form predictions for error rates, latency distributions, or threshold-crossing probabilities. In contrast, sequential analysis trades low-level detail for analytical transparency; it provides exact or asymptotic expressions for these quantities with far lower computational cost. Its value is therefore complementary rather than competitive: classical models capture device realism, while sequential analysis supplies principled performance bounds and interpretable operating points that would otherwise require extensive simulation to estimate.

The same threshold-centric view also connects to contemporary SNN architectures. In models such as Spiking Transformers (Zhao et al., 2025), multi-modal SNNs with temporal attention (Shen et al., 2025), or recurrent SNNs employing adaptive history mechanisms (Xu et al., 2023), performance ultimately depends on how local membrane-state variables cross internal thresholds to emit spikes. Sequential analysis provides closed-form links between increment statistics, firing probabilities, and expected latencies. These metrics can inform the co-design of thresholds or attention-gating rules in these architectures. More biologically grounded models with adaptive dendritic processes (Mao et al., 2025) can also be framed in this way: the dendritic dynamics shape the effective distribution seen at the soma (or the relevant spike initiation zone), and the same machinery characterizes the resulting crossing statistics. Thus, while specific implementation details differ across architectures, the underlying principles–increment distributions, drift, variance, and threshold geometry–admit the same analytical treatment and offer a complementary tool for understanding and tuning complex SNNs.

Classical sequential tests require exact likelihood functions to define optimal decision rules (Singh and Zhurbenko, 1975; Bartoo and Puri, 1967). The assumption of known likelihood ratios is fragile because its underlying distributions may be analytically intractable. When these functions are unknown, one approach is to exploit the Karlin–Rubin theorem. We can still identify uniformly most powerful tests within exponential families, even without explicit forms of the likelihood ratio. More generally, sequential analysis can be extended to composite hypothesis testing, where decision rules are constructed across a family of possible distributions rather than a single known model (Tartakovsky et al., 2014). We can often relax the requirement of closed-form likelihoods while retaining the sequential structure of accumulating evidence toward a threshold.

Sequential analysis provides more than a narrow mathematical idealization. We consider it a practical language for thinking about neuromorphic circuits. As a **benchmark**, it defines rigorous statistical baselines that reveal when hardware deviates from expectation. As a **proxy model**, it makes intractable circuit dynamics interpretable by recasting them in a simplified but faithful statistical form. As a **design tool**, it translates performance goals into circuit parameters, providing constructive prescriptions for circuit design rather than empirical tuning. While its classical formulation relies on restrictive assumptions of stationarity, independence, and closed-form likelihoods, these are not fatal obstacles. Extensions such as time-rescaling, correlated likelihoods, and composite hypothesis testing preserve the sequential paradigm while broadening its reach to realistic neuromorphic signals and circuits.

## Data Availability

The original contributions presented in the study are included in the article/Supplementary material, further inquiries can be directed to the corresponding author.
