# Supplementary material for: Sequential analysis and its applications to neuromorphic engineering
Source: Front Neurosci. 2026 Jan 9;19:1735027. doi: 10.3389/fnins.2025.1735027 (PMC12827506; doi:10.3389/fnins.2025.1735027)
Supplement: Supplementary file 1 [file Data_Sheet_1.pdf]

Supplementary: Sequential analysis and its applications to neuromorphic engineering

Shivaram Mani [1,*], **Saeed Afshar** [1] **and Travis Monk** [1]

[1] *International Centre for Neuromorphic Systems, The MARCS Institute, Western Sydney University, Sydney, NSW, Australia*

Correspondence*:
Shivaram Mani
S.Mani@westernsydney.edu.au

STATISTICS PREREQUISITES

This supplementary material reviews requisite statistics identities and concepts that we use in the main paper. We assume familiarity with expectations and conditional expectations; readers unfamiliar with conditional expectations can find a nice overview in Grimmett and Stirzaker (2020).

The law of total expectation

Let X and Y be random variables that live in the same probability space (i.e. under a common probability measure). Let $\mathbb{E}[X]$ be well-defined and $\mathbb{E}\left[X \middle| Y\right]$ be integrable. Then the law of total expectation says that:

$$\mathbb{E}\left[\mathbb{E}\left[X \middle| Y\right]\right] = \mathbb{E}[X].$$

An informal proof of this identity is straightforward and intuitive. The conditional expectation $\mathbb{E}\left[X \middle| Y\right]$ is a random variable whose value depends on the observation of Y (i.e. consider it as a function of Y). If X and Y only assume discrete values, then:

$$\begin{aligned}
\mathbb{E}\left[\mathbb{E}\left[X \middle| Y\right]\right] &= \mathbb{E}\left[\sum_x x \Pr(X = x|Y)\right] \\
&= \sum_y \left[\sum_x x \Pr(X = x|Y)\right] \Pr(Y = y) \\
&= \sum_x x \sum_y \Pr(X = x, Y = y) \\
&= \sum_x x \Pr(X = x) = \mathbb{E}[X].
\end{aligned}$$

This informal proof extends naturally to cases where X, Y, or both assume a continuous range of values. We simply replace the sums with integrals Grimmett and Stirzaker (2020).

This law allows us to 'split' an expectation into constituent conditional expectations. For example, say that Y can only take one of two values y_1 and y_2 with probabilities $\Pr(Y = y_1)$ and $\Pr(Y = y_2)$. Then we

can split $\mathbb{E}[X]$:

$$\begin{aligned}\mathbb{E}[X] &= \mathbb{E}\big[\mathbb{E}\big[X\big|Y\big]\big] \\ &= \mathbb{E}\big[X\big|Y = y_1\big]\Pr(Y = y_1) + \mathbb{E}\big[X\big|Y = y_2\big]\Pr(Y = y_2).\end{aligned}$$

Moment generating functions

Moment generating functions (MGFs) are an alternative description of a probability distribution. The MGF ϕ of a random variable X is defined as:

$$\phi_X(h) = \mathbb{E}\Big[e^{hX}\Big],$$

where h is an independent variable defined on the real numbers, $h \in \mathbb{R}$. MGFs exist for probability distributions that converge sufficiently fast to zero such that the sum or integral implicit in the expectation converges Billingsley (2017). If $\phi_X(h)$ exists, then there must be a unique probability distribution $\Pr(X)$ that corresponds to it Billingsley (2017); Feller (1991).

Writing out the expectation as an integral, we see that the MGF is effectively the Laplace transform of a probability distribution:

$$\phi_X(h) = \mathbb{E}\Big[e^{hX}\Big] = \int e^{hx}\Pr(X = x)dx.$$

Like the Laplace transform, the MGF transforms convolutions into products. This property is very useful in problems involving sums of independent random variables. For example, consider the sum $Z = X + Y$ where X and Y are independent. The distribution of Z is a convolution (Billingsley (2017)):

$$\Pr(Z = z) = \int \Pr(X = x)\Pr(Y = z - x)dx.$$

Depending on the forms of the distributions in the integrand, it might be impossible to analytically evaluate the distribution of Z. But the MGF of Z is simply the product of the MGFs of X and Y:

$$\begin{aligned}\phi_Z(h) = \mathbb{E}\Big[e^{hZ}\Big] &= \mathbb{E}\Big[e^{h(X+Y)}\Big] = \mathbb{E}\Big[e^{hX}e^{hY}\Big] \\ &= \mathbb{E}\Big[e^{hX}\Big]\mathbb{E}\Big[e^{hY}\Big] = \phi_X(h)\phi_Y(h),\end{aligned}$$

where we used the fact that $\mathbb{E}[XY] = \mathbb{E}[X]\mathbb{E}[Y]$ when X and Y are independent. Our complicated convolution in ‘distribution space’ becomes a product in ‘MGF space.’

We can quickly extend this property to finding the MGF of the sum of an arbitrary number of independent random variables. Let Z be the sum of N independent and identical random variables X, i.e. $Z = NX$. Then the MGF of Z becomes:

$$\phi_Z(h) = \phi_X(h)^N.$$

Furthermore, the MGF of a linear transformation of a random variable is easy to calculate. Let $Z = aX + b$, where a and b are constants:

$$\begin{aligned}\phi_Z(h) &= \mathbb{E}\Big[e^{hZ}\Big] = \mathbb{E}\Big[e^{haX}e^{hb}\Big]\\ &= \mathbb{E}\Big[e^{(ha)X}\Big]e^{hb} = \phi_X(ah)e^{hb}.\end{aligned}$$

The MGF gets its name from the fact that we can extract the moments of X from it. Taylor expanding the exponential:

$$\begin{aligned}\mathbb{E}\Big[e^{hX}\Big] &= \mathbb{E}\big[1 + hX/1! + h^2X^2/2! + \cdots\big]\\ &= 1 + h\mathbb{E}[X] + h^2\mathbb{E}\big[X^2\big]/2! + \cdots .\end{aligned}$$

We can find the kth moment of X by taking the kth derivative of $\phi_X(h)$ and evaluating it at $h = 0$:

$$\mathbb{E}\Big[X^k\Big] = \frac{d^k}{dh^k}\phi_X(h)\bigg|_{h=0}.$$

Characteristic functions

Characteristic functions (CFs) are another alternative description of a probability distribution. The CF ψ of a random variable X is defined as:

$$\psi_X(\tau) = \mathbb{E}\Big[e^{\tau X}\Big],$$

where τ is an independent variable that is purely imaginary, $\tau \in \mathbb{I}$.

The only difference between CFs and MGFs is the domain of their independent variables $\tau \in \mathbb{I}$ and $h \in \mathbb{R}$. This difference guarantees that $\psi_X(\tau)$ will always exist, i.e. the sum or integral implicit in the expectation always converges Billingsley (2017); Feller (1991). Writing out the expectation as an integral, we see that the CF is effectively the Fourier transform of a probability distribution:

$$\psi_X(\tau) = \mathbb{E}\Big[e^{\tau X}\Big] = \int e^{\tau X}\,\Pr(X = x)dx.$$

CFs enjoy the same convenient properties as MGFs. They also transform convolutions into products, so if $Z = X + Y$ where X and Y are independent, then:

$$\psi_Z(\tau) = \psi_X(\tau)\psi_Y(\tau).$$

If we sum N independent and identical random variables, i.e. $Z = NX$, then the CF of Z is:

$$\psi_Z(\tau) = \psi_X(\tau)^N.$$

If Z is a linear transformation of X, i.e. $Z = aX + b$, then the CF of Z is:

$$\psi_Z(\tau) = \psi_X(a\tau)e^{b\tau}.$$

We can also extract the moments of X from its CF:

$$\mathbb{E}\left[X^k\right] = i^{-k} \frac{d^k}{d\tau^k} \psi_X(\tau)\bigg|_{\tau=0}.$$

Unlike MGFs, CFs enjoy an existence-uniqueness theorem. Any admissible distribution of X uniquely specifies its CF, and vice-versa Billingsley (2017); Sobczyk and Kirkner (2001). So we can find the distribution of X from its CF via the inverse Fourier transform:

$$\Pr(X) = \frac{1}{2\pi} \int_{\mathbb{R}} e^{\tau T} \psi_X(\tau) d\tau.$$

Martingales

Let S_t be a stochastic process that occupies some state S at time t. For example, S_t could represent the randomly-fluctuating voltage of an event sensor pixel at time t. Say we can find some function f such that:

$$\mathbb{E}\big[f(S_t)\big|S_{t-1}\big] = f(S_{t-1}). \tag{1}$$

Then we say we have a martingale Grimmett and Stirzaker (2020); Williams (1991).

Martingales are the stochastic process analog to conservation laws in physics Kolsrud (2007); Roldán et al. (2023). In classical mechanics, we invoke the conservation of energy to find the trajectory of a particle in a conserved system over time. Kirchhoff's laws invoke conservation of charge and energy to find currents and voltages across electrical components in a circuit. Martingales are a statement that an expectation is conserved throughout a stochastic process. We show this conservation by taking the expectation of both sides of Eq. 1 and applying the law of total expectation:

$$\mathbb{E}\big[\mathbb{E}\big[f(S_t)\big|S_{t-1}\big]\big] = \mathbb{E}[f(S_t)] = \mathbb{E}[f(S_{t-1})].$$

So by induction:

$$\mathbb{E}[f(S_t)] = \mathbb{E}[f(S_0)].$$

Often in stochastic processes we assume that we know the value of a stochastic process at its beginning. For example, we could assume that the voltage of the event sensor pixel begins its stochastic trajectory at some known distance from its on and off thresholds. Then $f(S_0)$ is not random:

$$\mathbb{E}[f(S_t)] = f(S_0).$$

If we find a martingale for some f, then we know $\mathbb{E}[f(S_t)]$ at all future times because the expectation is conserved.

Doob's optional stopping theorem states that a randomly-stopped martingale is still a martingale Bhattacharya and Waymire (2007); Grimmett and Stirzaker (2020). For example, the time that an event sensor pixel's voltage crosses a threshold can be considered as a random variable T. Doob says that we can insert a random variable T for t:

$$\mathbb{E}[f(S_T)] = f(S_0),$$

as long as $f(S_t)$ is bounded and integrable:

$$\|f(S_t\| < M \quad \forall t, M \in \mathbb{R} \quad \text{and} \quad \mathbb{E}[f(S_t)] < \infty \quad \forall t$$

Martingales are very powerful tools to study stochastic processes. We can often extract elegant expressions for their global statistics from a martingale, if we can find one.

REFERENCES

Bhattacharya, R. N. and Waymire, E. C. (2007). *A basic course in probability theory*, vol. 69 (Springer)

Billingsley, P. (2017). *Probability and measure* (John Wiley & Sons)

Feller, W. (1991). *An introduction to probability theory and its applications, Volume 2*, vol. 2 (John Wiley & Sons)

Grimmett, G. and Stirzaker, D. (2020). *Probability and random processes* (Oxford university press)

Kolsrud, T. (2007). Quantum and classical conserved quantities: Martingales, conservation laws and constants of motion. In *Stochastic Analysis and Applications: The Abel Symposium 2005* (Springer), 461–491

Roldán, É., Neri, I., Chetrite, R., Gupta, S., Pigolotti, S., Jülicher, F., et al. (2023). Martingales for physicists: a treatise on stochastic thermodynamics and beyond. *Advances in Physics* 72, 1–258

Sobczyk, K. and Kirkner, D. J. (2001). *Stochastic modeling of microstructures* (Springer Science & Business Media)

Williams, D. (1991). *Probability with martingales* (Cambridge university press)
